# A nested case–control study of risk for pulmonary embolism in the general trauma population using nationwide trauma registry data in Japan

**DOI:** 10.1038/s41598-021-98692-4

**Published:** 2021-09-28

**Authors:** Hiroki Iriyama, Akira Komori, Takako Kainoh, Yutaka Kondo, Toshio Naito, Toshikazu Abe

**Affiliations:** 1 Department of General Medicine, Faculty of Medicine, Juntendo University, 2-1-2 Hongo, Bunkyo-ku, Tokyo 113-8421 Japan; 2 Department of Emergency and Critical Care Medicine, Juntendo University Urayasu Hospital, 2-1-1 Tomioka, Urayasu, Chiba 270-0021 Japan; 3 Department of Health Services Research, Faculty of Medicine, University of Tsukuba, 1-1-1 Tennodai, Tsukuba, Ibaraki 305-8577 Japan; 4 Health Services Research and Development Center, University of Tsukuba, 1-1-1 Tennodai, Tsukuba, Ibaraki 305-8577 Japan; 5 Department of Emergency and Critical Care Medicine, Tsukuba Memorial Hospital, 1187-299 Kaname, Tsukuba, Ibaraki 300-2622 Japan

**Keywords:** Risk factors, Embolism

## Abstract

Post-trauma patients are at great risk of pulmonary embolism (PE), however, data assessing specific risk factors for post-traumatic PE are scarce. This was a nested case–control study using the Japan Trauma Data Bank between 2004 and 2017. We enrolled patients aged ≥ 16 years, Injury Severity Score ≥ 9, and length of hospital stay ≥ 2 days, with PE and without PE, using propensity score matching. We conducted logistic regression analyses to examine risk factors for PE. We included 719 patients with PE and 3595 patients without PE. Of these patients, 1864 [43.2%] were male, and their median Interquartile Range (IQR) age was 73 [55–84] years. The major mechanism of injury was blunt (4282 [99.3%]). Median [IQR] Injury Severity Score (ISS) was 10 [9–18]. In the multivariate analysis, the variables spinal injury [odds ratio (OR), 1.40 (1.03–1.89)]; long bone open fracture in upper extremity and lower extremity [OR, 1.51 (1.06–2.15) and OR, 3.69 (2.89–4.71), respectively]; central vein catheter [OR, 2.17 (1.44–3.27)]; and any surgery [OR, 4.48 (3.46–5.81)] were independently associated with PE. Spinal injury, long bone open fracture in extremities, central vein catheter placement, and any surgery were risk factors for post-traumatic PE. Prompt initiation of prophylaxis is needed for patients with such trauma.

## Introduction

Pulmonary embolism (PE) poses a great risk of mortality and morbidity for patients after trauma^1,2^, among whom prophylaxis for PE is an important consideration. However, patients with lower extremity trauma were unable to receive mechanical prophylaxis. Neither could pharmacologic prophylaxis be applied to patients with high risk of bleeding—especially soon after trauma or surgery. It is thus important to carefully assess risk of PE for each individual patient.

Previous studies evaluated the risk of post-traumatic PE. Aging^3–10^ and trauma severity^5–9,11–14^ are established risk factors. Other, more controversial risk factors of post-traumatic PE are injury sites, blood transfusion, and timing of definitive surgery. Previous studies were not designed to assess specific risk factors for post-traumatic PE, rather investigating the overall outcome of venous thromboembolism (VTE), including deep vein thrombosis (DVT). Few risk assessment studies have focused only on post-traumatic PE. Patients with post-traumatic PE, known as de novo PE, do not often have DVT. The risk of PE and DVT may be different among patients with trauma^15–17^.

We therefore aimed to investigate specific unknown risk factors for acute PE such as injury sites and timing of definitive surgery in trauma patients using a nationwide trauma registry in Japan.

## Methods

### Design and setting

We conducted a nested case–control study between January 2004 and December 2017 utilizing a nationwide trauma registry, the Japan Trauma Data Bank (JTDB) database, established in 2003. In 2017, a total of 264 hospitals, including 95% of all tertiary emergency medical centers in Japan, participated in the database^18^, which includes patient demographics, Abbreviated Injury Scale (AIS) scores, Injury Severity Score (ISS), emergency procedures, in-hospital complications, and clinical outcomes. Data collection was performed as a part of routine clinical patient management.

### Participants

**Figure 1:**
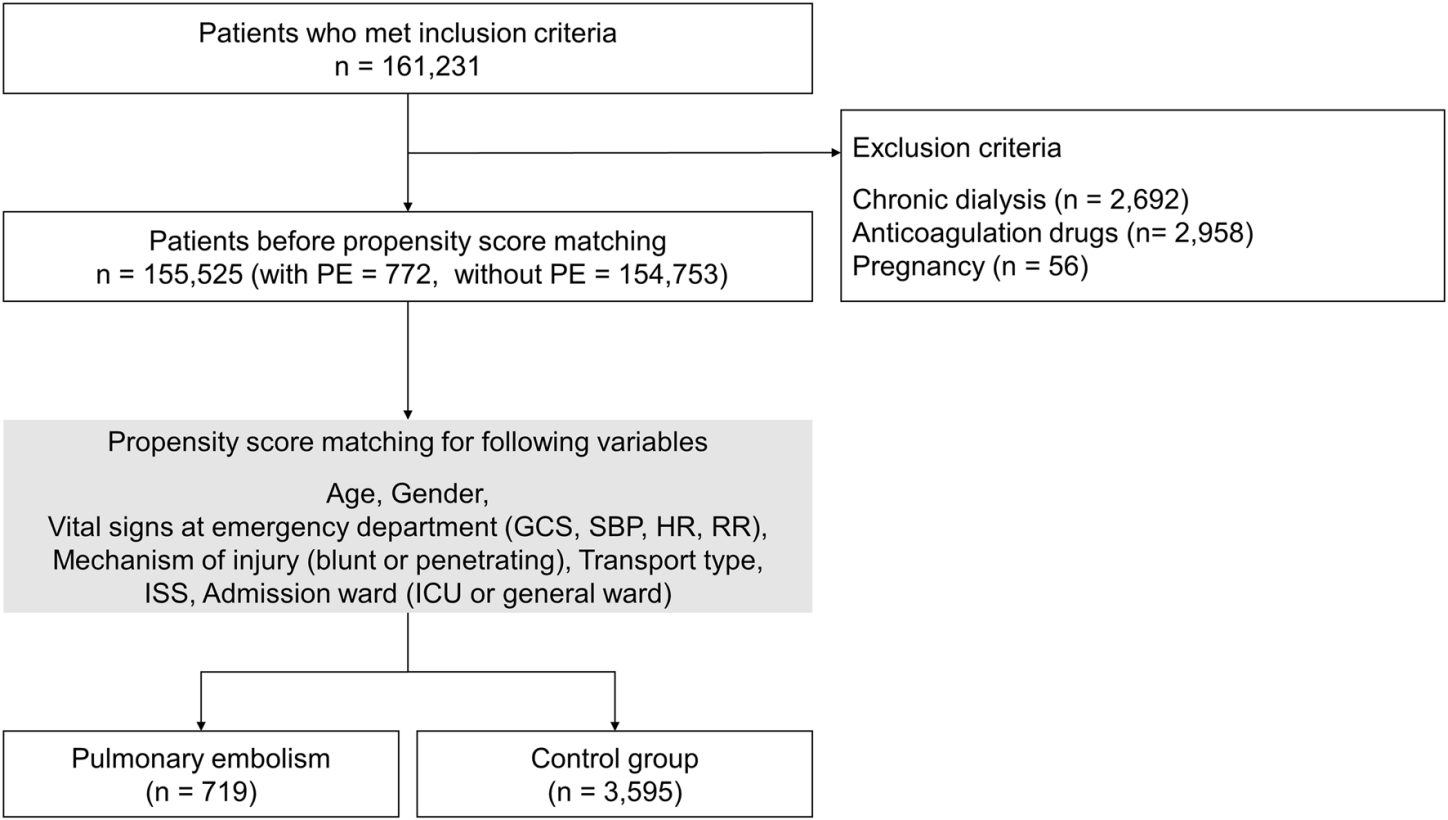
Patient selection. Definition of abbreviations: PE = pulmonary embolism; ISS = Injury Severity Score; GCS = Glasgow Coma Scale; SBP = systolic blood pressure; HR = heart rate; RR = respiratory rate; ICU = intensive care unit.

We included patients who had blunt or penetrative trauma, were aged ≥ 16 years old, and were admitted to the intensive care unit (ICU) or general ward. We included only patients who had ISS ≥ 9 because PE is rare among patients with ISS < 9^8,13^, which is the same method in previous studies^13,14,19–21^. We included only patients who survived for more than 2 days after hospital admission in order to exclude the impact of early trauma deaths^14,22^. We excluded patients who were pregnant, underwent chronic dialysis^20^, and received anticoagulation drugs (Fig. 1).

### Data definitions

The primary outcome was occurrence of PE during hospital stay. The diagnosis of PE was based on the reports of the physician in charge. The database has no data on diagnostic measure. We categorized AIS codes representing fractures into open, closed, or unclassifiable, using AIS 90 Update 98 and AIS 2005 Update 2008. We categorized AIS codes such as 752,604.3 (humerus fracture), 752,804.3 (radius fracture), 753,204.3 (ulna fracture), 853,422.3 (tibia fracture), and 852,604.3 (pelvic fracture) in AIS 90 Update 98 as open fractures in this study because almost all were open, although these codes include several other kinds of fractures. We defined blood transfusion as any blood product within the first 24 h—the same method as that used in previous studies^20,23,24^. The database recorded central vein catheter placements only at the emergency department.

### Statistical analyses

We included patients from the database without PE as a control, using propensity score (PS) matching to ensure that the PE group and the control group were equally balanced on baseline characteristics and severity of trauma. We used the variables age, gender, vital signs at emergency department (Glasgow Coma Scale, systolic blood pressure, heart rate, and respiratory rate), mechanism of injury, transport type, ISS, and admission ward, to calculate PS. We carefully selected these variables based on clinical relevance and previous research^18,25^. We employed nearest neighbor matching without replacement in a 1:5 manner. We used a caliper of 0.01 standard deviation of the logit of the PS. We evaluated standardized mean difference of the variables over 0.1 as a meaningful imbalance after PS matching. We calculated descriptive statistics comparing the PE group and the control group, using the Wilcoxon signed-rank test for continuous variables and the chi-square or Fisher exact test for categorical variables, having first evaluated the normality of all continuous variables using the Kolmogorov–Smirnov test. No continuous variables had normal distributions.

We used conditional logistic regression models to identify specific risk factors for post-traumatic PE. In the 1st model, the candidates of risk factors were body regions with AIS ≥ 3. In the 2nd model we included long bone and pelvic fracture instead of AIS ≥ 3 in the upper and lower extremities. In the 3rd model, we categorized long bone and pelvic fracture into open, closed, or unclassifiable fractures. In the final model, we added procedures such as blood transfusion, central vein catheter placement, and any surgery, to the 3rd model. We adjusted all the models for comorbidities (see Supplementary Table S1). We did not adjust all the models for in-hospital complications such as pneumonia and sepsis because the database had no data on whether PE or these in-hospital complications occurred antecedently. For all models we did not use covariates which had 10 or less patients in the PE group or control group. When we conducted conditional logistic regression models, we calculated the variance inflation factor of the covariates to evaluate multicollinearity, evaluating a variance inflation factor > 5 as meaningful multicollinearity. However, we did not confirm any meaningful multicollinearity. All *p* values were two-sided; we considered *p* values of < 0.05 statistically significant. We performed statistical analyses using R software (Version 3.6.2)^26^.

### Subgroup analysis

We conducted a subgroup analysis to investigate the influence of time to bone fixation. We included only patients who had received primary bone fixation. We used a non-conditional logistic regression model with covariates time to bone fixation and the same covariates as those used in the final model. We defined time to bone fixation as the time between hospital arrival and surgery. We did not use covariates which had 10 or less patients in the PE group or control group for the model, in the same way as in the main analysis.

### Sensitivity analysis

We conducted a sensitivity analysis to exclude the possibility that mechanical and pharmacological prophylaxis could be potential confounding elements. Because prophylaxis is well adhered to in ICU in Japan, we included only patients admitted to the ICU. We used a non-conditional logistic regression model and included the same covariates as those in the final model. Again, we did not use covariates which had 10 or less patients in the PE group or the control group for the model, using the same method as in the main analysis.

### Imputation

Before PS matching, we replaced missing values concerning vital signs at the emergency department with vital signs at the injury site (we replaced no missing records for the Glasgow Coma Scale, 18 missing for systolic blood pressure, 82 missing for heart rate, and 318 missing for respiratory rate among all the 4314 patients in this study). We defined missing values for blood transfusion as no blood transfusion (82 missing among the 4314 patients in this study). We replaced missing values for time of hospital arrival with time when an emergency medical technician had contacted patients at the injury site (67 missing among 1997 patients who received primary bone fixation).

### Ethics approval

The Ethics Committee of the Juntendo University approved this study (IRB No. 19-010). We confirmed that all methods were performed in accordance with the relevant guidelines. We confirmed that the need for informed consent was waived by The Ethics Committee of the Juntendo University. The JTDB administrators also provided permission for use of the data from their database.

### Ethics approval and consent to participate

The Ethics Committee of the Juntendo University, which did not mandate obtaining consent from patients in observational studies using anonymous data, approved this study (IRB No. 19-010).

## Results

A total of 155,525 patients among the patients who were registered in the JTDB database were eligible for this study after inclusion and exclusion criteria. A total of 772 (0.5%) patients developed post-traumatic PE. After PS matching, the PE group comprised 719 patients and the control group comprised 3595 patients (Fig. 1). We detected no meaningful imbalance of the variables for the PS matching between the two groups (Table 1).

**Table 1:** Baseline characteristics of patients with and without pulmonary embolism.

Characteristics		PE group (n = 719)	Control group (n = 3595)	SMD	*p* value
Age, median (IQR)^a^		74 (55–84)	73 (56–84)	0.008	
Male, No (%)^a^		321 (44.6)	1543 (42.9)	0.04	
Mechanism of injury, No (%)^a^	Blunt/Penetrate	713/6 (99.2/0.8)	3569/26 (99.3/0.7)	0.01	
Transport type, No (%)^a^	Ambulance without physician	647 (90.0)	3205 (89.2)	0.04	
	Ambulance/Helicopter with physician	58 (8.1)	296 (8.2)		
	Other	14 (1.9)	94 (2.6)		
Vital signs at ED, median (IQR)^a^	GCS	15 (14–15)	15 (14–15)	0.005	
	SBP	140 (119–159)	140 (120–161)	0.04	
	HR	82 (71–95)	82 (72–95)	0.02	
	RR	20 (16–24)	20 (18–24)	0.009	
Admission ward, No (%)^a^	General ward/ICU	393/326 (54.7/45.3)	2011/1584 (55.9/44.1)	0.03	
ISS, median (IQR)^a^		10 (9–20)	10 (9–18)	0.02	
AIS ≥ 3, No (%)	Head	119 (16.6)	1050 (29.2)		< 0.001
	Thorax	137 (19.1)	768 (21.4)		0.18
	Abdomen	46 (6.4)	194 (5.4)		0.33
	Spine	75 (10.4)	387 (10.8)		0.84
	Upper extremity	59 (8.2)	186 (5.2)		0.002
	Lower extremity (including pelvis)	479 (66.6)	1690 (47.0)		< 0.001
Upper extremity (detail), No (%)	Long bone fracture	60 (8.3)	187 (5.2)		0.001
	Open	57 (7.9)	165 (4.6)		< 0.001
	Closed	3 (0.4)	23 (0.6)		0.61
Lower extremity and pelvis (detail), No (%)	Long bone and pelvic fracture	490 (68.2)	1670 (46.5)		< 0.001
	Open	199 (27.7)	276 (7.7)		< 0.001
	Closed	56 (7.8)	239 (6.6)		0.31
	Unclassifiable^b^	283 (39.4)	1306 (36.3)		0.14
Blood transfusion, No (%)		132 (18.4)	511 (14.2)		0.006
Central vein catheter, No (%)		50 (7.0)	105 (2.9)		< 0.001
Surgery, No (%)	**Any**	623 (86.6)	2051 (57.1)		< 0.001
	Orthopedic	550 (76.5)	1650 (45.9)		< 0.001
	Bone fixation	521 (72.5)	1476 (41.1)		< 0.001
	Abdominal	48 (6.7)	181 (5.0)		0.09
	Head	32 (4.5)	184 (5.1)		0.51
	Chest	11 (1.5)	34 (0.9)		0.16
	Others^c^	23 (3.2)	103 (2.9)		0.72

Missing data: None.

*PE* pulmonary embolism, *SMD* standardized mean difference, *IQR* interquartile range, *ED* emergency department, *GCS* Glasgow Coma Scale, *SBP* systolic blood pressure, *HR* heart rate, *RR* respiratory rate, *ICU* intensive care unit, *ISS* Injury Severity Score, *AIS* abbreviated injury scale score.

^a^Variables included in the propensity score matching.

^b^AIS90 Update 98 included lower extremity and pelvic fracture codes which represent both open and closed fracture.

^c^Other surgeries include facial, neck, and skin surgeries.

With regard to baseline characteristics, the PE group had head injury with AIS ≥ 3 less frequently and lower extremities and pelvic injury with AIS ≥ 3 more frequently than those of the control group (16.6% vs. 29.2%, 66.6% vs. 47.0%, respectively) (Table 1). Specifically, the PE group had long bone and open fractures in the upper and lower extremities more frequently than the control group. We found no consistent pattern of comorbidities between the two groups (e-Table 1). However, we noted several obvious concomitant complications that occurred more frequently in the PE group than in the control group (see Supplementary Table S2). Such complications included pneumonia (47.4% vs. 2.9%), osteomyelitis (44.6% vs. 0.1%), sepsis or multiple organ failure (30.7% vs. 0.6%), and wound infection (51.5% vs. 1.1%).

Regarding treatments, the PE group frequently received blood transfusion (18.4% vs. 14.2%, *p* = 0.006), central vein catheter placement (7.0% vs. 2.9%, *p* < 0.001), and surgery (86.6% vs. 57.1%, *p* < 0.001). In both groups, bone fixation was the most frequent surgery (72.5% and 41.4%). Among patients who received bone fixation, a lower proportion of the PE group than that of the control group received bone fixation within 24 h (22.4% vs. 27.5%) (see Supplementary Table S3).

The overall in-hospital mortality was higher in the PE group than in the control group (5.8% vs. 3.4%, *p* = 0.003) (Table 2). In terms of survivor dispositions, the PE group were more likely to be transferred to other facilities than the control group (68.9% vs. 58.4%, *p* < 0.001). The PE group had longer hospital stays than the control group (32 [interquartile range (IQR): 19–60] days vs. 23 [IQR: 13–36] days, *p* < 0.001).

**Table 2:** Outcomes of patients with and without pulmonary embolism.

Outcomes	PE group (n = 719)	Control group (n = 3595)	*p* value
In-hospital mortality, No (%)	42 (5.8)	122 (3.4)	0.003
**Place after discharge, No (%)**			
Home	204 (30.2)	1365 (39.7)	< 0.001
Other facilities	466 (68.9)	2006 (58.4)	
Other	6 (0.9)	64 (1.9)	
Length of hospital stay, median (IQR)	32 (19–60)	22 (13–36)	< 0.001

Missing data: In-hospital mortality = 32; Place after discharge = 203.

*PE* pulmonary embolism, *IQR* interquartile range.

Table 3 shows conditional logistic regression models to identify risk factors for PE. Head injury with AIS ≥ 3 was associated with a lower occurrence of PE in the 1st, 2nd, and 3rd model. However, in the final model, head injury was not associated with PE (odds ratio (OR) [95% confidence interval (CI)]: 0.78 [0.61–1.01]). Orthopedic injury such as spine injury with AIS ≥ 3 (OR [95% CI]: 1.40 [1.03–1.89]), open fracture in the upper extremities (OR [95% CI]: 1.51 [1.06–2.15]), and open fracture in the lower extremities (OR [95% CI]: 3.69 [2.89–4.71]) were independently associated with PE in the final model. Regarding treatments, central vein catheter placement (OR [95% CI]: 2.17 [1.44–3.27]) and any surgery (OR [95% CI]: 4.48 [3.46–5.81]) were associated with PE in the final model.

**Table 3:** Association between pulmonary embolism and various characteristics from the models under consideration.

Variable	OR (95% CI)			
	1st model (n = 4314)	2nd model (n = 4314)	3rd model (n = 4314)	Final model (n = 4314)
Head AIS ≥ 3	0.77 (0.60–0.98)	0.76 (0.60–0.97)	0.67 (0.53–0.86)	0.78 (0.61–1.01)
Thorax AIS ≥ 3	1.20 (0.95–1.52)	1.13 (0.90–1.42)	1.09 (0.87–1.38)	1.22 (0.95–1.56)
Abdomen AIS ≥ 3	1.48 (1.03–2.11)	1.47 (1.03–2.11)	1.42 (0.99–2.04)	1.00 (0.68–1.48)
Spine AIS ≥ 3	1.50 (1.12–2.02)	1.51 (1.13–2.02)	1.26 (0.94–1.69)	1.40 (1.03–1.89)
Upper extremity AIS ≥ 3	2.26 (1.62–3.16)			
**Long bone fracture**		2.27 (1.63–3.15)		
Open			2.24 (1.59–3.15)	1.51 (1.06–2.15)
Closed			^a^	^a^
Lower extremity AIS ≥ 3	2.61 (2.08–3.26)			
**Long bone and pelvic fracture**		2.79 (2.25–3.46)		
Open			4.82 (3.82–6.10)	3.69 (2.89–4.71)
Closed			1.18 (0.85–1.63)	1.03 (0.73–1.44)
Blood transfusion				0.84 (0.64–1.09)
Central vein catheter				2.17 (1.44–3.27)
Any Surgery				4.48 (3.46–5.81)

All the models were also controlled for comorbidities.

The 3rd model and the final model were adjusted for unclassifiable fracture in lower extremity and pelvis.

*OR* odds ratio, *CI* confidence interval, *AIS* Abbreviated Injury Scale score.

^a^Covariate which was not used for the model because of less than 10 patients in PE group.

**Figure 2:**
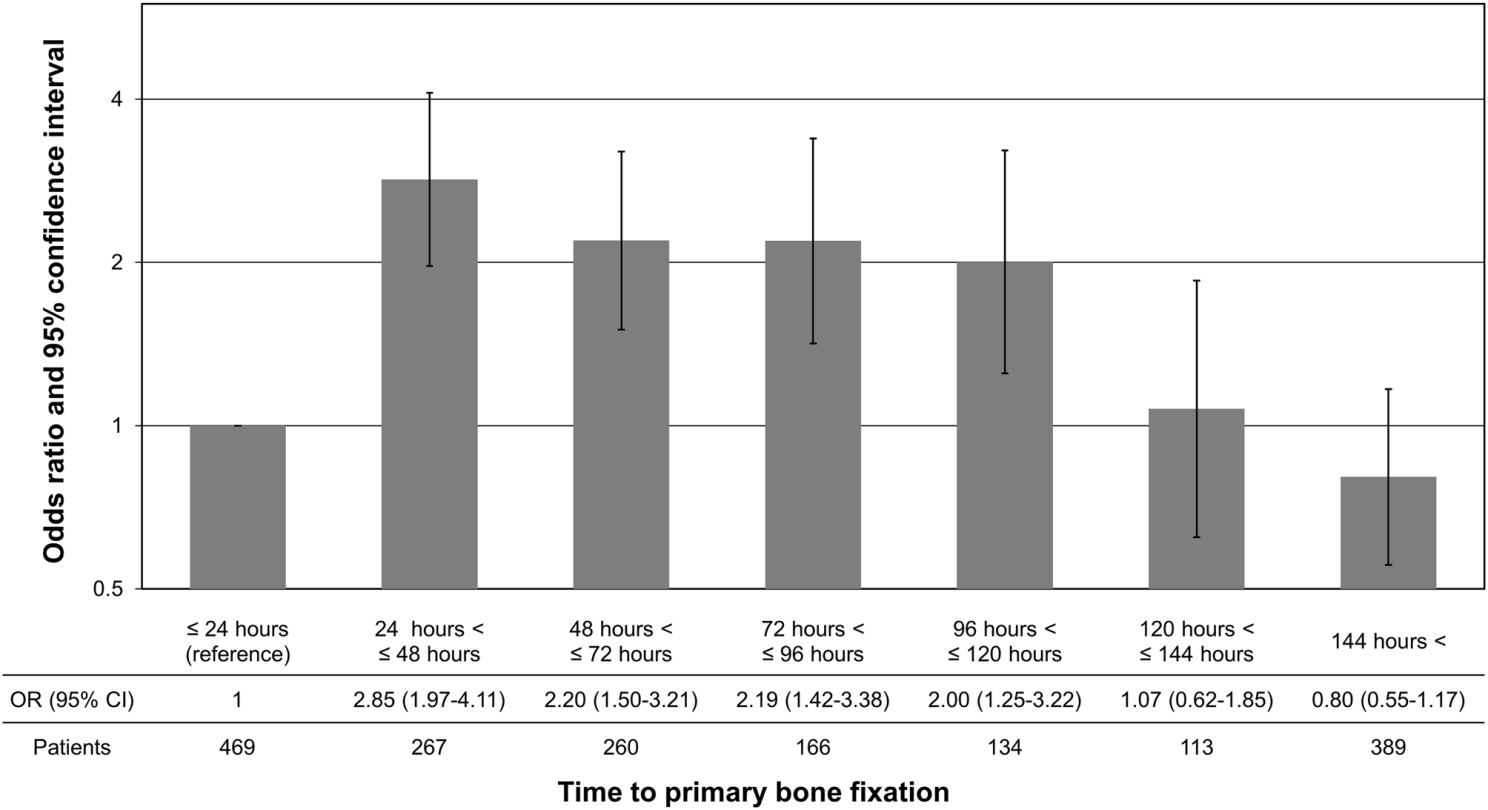
Association between pulmonary embolism and time to primary bone fixation. This figure is the result of subgroup analysis using non-conditional logistic regression model. This model was adjusted for the same covariates as in the final model. Definition of abbreviations: OR = odds ratio; CI = confidence interval.

In the subgroup analysis, we included 1997 patients treated with primary bone fixation. Our non-conditional logistic regression model showed that time to primary bone fixation of 24–120 h—compared to that within 24 h—was associated with PE (Fig. 2).

Sensitivity analysis showed that the following covariates were independently associated with PE, which is consistent with the final model: spine AIS ≥ 3 (OR [95% CI]: 1.62 [1.15–2.27]); open fracture in lower extremity (OR [95% CI]: 2.11 [1.50–2.96]); central vein catheter placement (OR [95% CI]: 1.91 [1.27–2.86]); and any surgery (OR [95% CI]: 3.53 [2.57–4.85]) (see Supplementary Table S4). Only Open fracture in the upper extremities was not associated with PE (OR [95% CI]: 1.30 [0.80–2.10]), a finding which differed from that of the final model.

## Discussion

This nested case–control study of general trauma population revealed that long bone open fracture in extremities, spinal injury, central vein catheter placement, and any surgery were risk factors for PE. Bone fixation was the most frequent surgery, and delayed bone fixation also could be a risk factor for PE. Among these risk factors, long bone open fracture in extremities and delayed bone fixation are novel findings added by the current research.

Long bone open fractures in upper and lower extremities emerged as high risk trauma for PE. Lower extremity fracture is an established risk factor for PE; however, in previous studies, upper extremity fracture has not been risk factor for PE^3,8,10,23^. Whether open or closed fracture was a risk factor for PE was also unknown. In this study, long bone open fracture in the lower extremities was the most relevant trauma with PE, and long bone open fracture in the upper extremities was the second most relevant trauma. The possible underlying pathophysiology may be that open fracture has greater soft tissue damage involving the venous endothelium and has more bleeding from open wounds, resulting in more severe coagulopathy, than closed fracture^27–29^. Venous stasis caused by immobility could also play a role in patients with lower extremity fracture^30^. Open fracture in the extremities could be related to occurrence of PE.

We confirmed that spinal injury^9,12,23,31–33^, central vein catheter placement^6,34,35^, and surgery^3,10,12,23^ were risk factors for PE, which is consistent with previous studies. Spinal injury is related to venous stasis because of long-term immobility. Vascular-related procedures such as central vein catheter placement and surgery could be related to endothelial injury and hypercoagulability.

Head, thoracic, and abdominal trauma were not risk factors for PE in this study, although whether these injury sites are risk factors for PE has been controversial in previous studies^3,4,7,9,11^. Compared with this study, previous studies were limited by small sample size and uncontrolled potential confounders such as comorbidities, severity, and surgery. Regarding head trauma, recent studies focusing on the timing of post-traumatic PE showed that head trauma was associated with late onset of PE^16,36–38^. Our cohort might have captured only early PE.

Early definitive surgery for bone fracture could reduce risk for PE. Previous studies showed that delayed definitive surgery for bone fracture (after 24 to 48 h) led to pulmonary complications such as pneumonia and acute respiratory distress syndrome because post-traumatic and post-surgical inflammation act as “two hit model”^24,39–41^. However, few studies have showed an association between timing of definitive surgery and post-traumatic PE because it is a relatively infrequent complication^42^. A subgroup analysis in this study showed that bone fixation during 24–120 h after injury was associated with a higher risk for PE. Our study suggests that early total care is better than damage control orthopedics regarding PE among patients with bone fracture.

In this study, patients with spinal injury, open fracture in the extremities, central vein catheter placement, and any surgery were at high risk of post-traumatic PE. Patients with these characteristics should be probably treated with pharmacological prophylaxis as soon as bleeding risk is adequately controlled.

The current study has several strengths. First, our study focused on specific risk factor for post traumatic PE such as open fracture and delayed definitive surgery by using PS matching. By PS matching, our cohort was well balanced in terms of general known risk factors for PE such as age, gender, and severity. Second, our study could have less sampling bias because our study is nationwide cohort study which almost all tertiary emergency centers in Japan participated in. Characteristics of PE in this study could be representative for generalizability of post traumatic PE. Third, we focused on preventable post traumatic PE by excluding the impact of early trauma deaths. There must be no lethal PE occurring just after trauma caused by trauma-induced hypercoagulability in this study.

The current study has several important limitations that warrant discussion. First, neither mechanical nor pharmacological prophylaxis was recorded in this database. It could be potential confounding factors. However, prophylaxis has been well adhered to in Japan since 2004 because of the following reasons: the primary domestic guideline of prevention of VTE was published in 2004; and the payment of management fee to hospitals for the prevention of VTE started in 2004^43^. Moreover, we performed a sensitivity analysis among ICU patients because prophylaxis has been well adhered to especially in ICU (see Supplementary Table S4). The sensitivity analysis showed similar results, four of five risk factors for PE in the main analysis were independently associated with PE. Second, because they were risk factors for PE in previous studies, pneumonia and sepsis also could be potential confounding factors^13,34,44^. However, we did not include these complications to logistic regression analyses because we did not have onset-time data of complications in our database. Third, we did not include patients who did not survive < 48 h to exclude the impact of early trauma deaths. Some patients with PE might have been lost in our study. However, because VTE prophylaxis such as anticoagulants could not have been used in early phase of trauma care, these patients were presumably small number and their deaths were unpreventable. Fourth, there might be an information bias. The diagnosis of PE was based on the reports of the physician in charge, and it might have been an underdiagnosis. However, the incidence of PE among patients with eligible criteria was 0.5% (772/155,525) in this study, which is consistent with previous studies (0.1–2.6%)^3,5–7,9–11,42^. In addition, physicians in Japan have easy access to the use of laboratory data such as D-Dimer, echocardiography, and echocardiography. Moreover, physicians in Japan, compared with those in other countries, have relatively easier access to the use of computed tomography, because there are many computed tomography scanners in Japan^45^.

## Conclusions

Among general trauma population, long bone open fracture in extremities, spinal injury, central vein catheter placement, and any surgery could be risk factors for post-traumatic PE. Prompt initiation of prophylaxis is needed for patients with such trauma. Among patients with bone fracture, delayed bone fixation also could be a risk factor for PE. Early definitive surgery for bone fracture could reduce risk for PE.

## Supplementary Information

Supplementary Tables.

## Data Availability

The datasets generated during and/or analysed during the current study are available from the corresponding author on reasonable request.
