# Supplementary material for: A nested case–control study of risk for pulmonary embolism in the general trauma population using nationwide trauma registry data in Japan
Source: Sci Rep. 2021 Sep 28;11:19192. doi: 10.1038/s41598-021-98692-4 (PMC8478977; doi:10.1038/s41598-021-98692-4)
Supplement: Supplementary file 1 — Supplementary Tables. [file 41598_2021_98692_MOESM1_ESM.docx]

**Supplementary information**

**Supplementary Table S1:** **Comorbidities of patients with and without pulmonary embolism**

|  | n (%) | |  | n (%) | |
| --- | --- | --- | --- | --- | --- |
| Comorbidities | **PE (n = 719)** | **Control (n = 3,595)** | **Comorbidities** | **PE (n = 719)** | **Control (n = 3,595)** |
| **Circulation** | | | **Metabolism** | | |
| Ischemic heart diseases | 51 (7.1) | 164 (4.6) | DM | 88 (12.2) | 440 (12.2) |
| Heart failure | 26 (3.6) | 118 (3.3) | Obesity | 3 (0.4) | 8 (0.2) |
| Hypertension | 233 (32.4) | 1170 (32.5) | Others | 12 (1.7) | 122 (3.4) |
| Others | 24 (3.3) | 182 (5.1) | **Neurological** | | |
| **Respiratory** | | | Stroke | 68 (9.5) | 241 (6.7) |
| Asthma | 29 (4.0) | 119 (3.3) | Psychiatric disease | 49 (6.8) | 178 (5.0) |
| COPD | 6 (0.8) | 25 (0.7) | Dementia | 97 (13.5) | 314 (8.7) |
| Others | 6 (0.8) | 37 (1.0) | Others | 9 (1.3) | 111 (3.1) |
| **Gastroenterology & hepatobiliary** | | | **Others** | | |
| Liver cirrhosis | 1 (0.1) | 21 (0.6) | HIV | 0 (0.0) | 1 (0.0) |
| Chronic hepatitis | 14 (1.9) | 48 (1.3) | Malignancies | 8 (1.1) | 93 (2.6) |
| Peptic ulcer | 19 (2.6) | 80 (2.2) | Hematological diseases | 0 (0) | 9 (0.3) |
| IBD | 2 (0.3) | 15 (0.4) | Steroid | 1 (0.1) | 16 (0.4) |
| Others | 15 (2.1) | 154 (4.3) | Immunosuppressant | 1 (0.1) | 5 (0.1) |
|  |  |  | Others | 17 (2.4) | 163 (4.5) |

Definition of abbreviations: PE = pulmonary embolism; COPD = chronic obstructive pulmonary disease; IBD = inflammatory bowel diseases; DM = diabetes mellitus; HIV = human immunodeficiency virus.

**Supplementary Table S2:** **Complications in patients with and without pulmonary embolism**

|  | n (%) | |  | n (%) | |
| --- | --- | --- | --- | --- | --- |
| Comorbidities | **PE (n = 719)** | **Control (n = 3,595)** | **Comorbidities** | **PE (n = 719)** | **Control (n = 3,595)** |
| **Central nerve system** | | | **Gastroenterology & hepatobiliary** | | |
| Diabetes insipidus | 2 (0.3) | 8 (0.2) | Ulcer/Upper GI bleeding | 10 (1.4) | 21 (0.6) |
| Hydrencephalus | 4 (0.6) | 6 (0.2) | Ileus | 11 (1.5) | 8 (0.2) |
| Fat embolism | 133 (18.5) | 2 (0.1) | Pancreatitis | 2 (0.3) | 2 (0.1) |
| CSF leakage | 2 (0.3) | 12 (0.3) | Cholecystitis | 9 (1.3) | 4 (0.1) |
| Meningitis | 7 (1.0) | 6 (0.2) | Bilirubinemia/Liver failure | 4 (0.6) | 5 (0.1) |
| Higher brain dysfunction | 16 (2.2) | 68 (1.9) | Others | 7 (1.0) | 27 (0.8) |
| Mental disorders | 12 (1.7) | 22 (0.6) | **Bone & joint** | | |
| Others | 25 (3.5) | 56 (1.6) | Compartment syndrome | 261 (36.3) | 6 (0.2) |
| **Circulation** | | | Osteomyelitis | 321 (44.6) | 4 (0.1) |
| Acute coronary syndrome | 17 (2.4) | 1 (0.0) | Refracture | 317 (44.1) | 4 (0.1) |
| Lethal arrhythmia | 3 (0.4) | 4 (0.1) | nonunion | 322 (44.8) | 1 (0.0) |
| Refractory shock | 4 (0.6) | 11 (0.3) | Others | 54 (7.5) | 18 (0.5) |
| CPA | 15 (2.1) | 21 (0.6) | **Infection et al.** | | |
| Acute kidney injury | 8 (1.1) | 12 (0.3) | Bacteremia | 19 (2.6) | 12 (0.3) |
| Abdominal compartment | 2 (0.3) | 0 (0) | Sepsis or MOF | 221 (30.7) | 20 (0.6) |
| Others | 31 (4.3) | 27 (0.8) | Abdominal abscess | 2 (0.3) | 5 (0.1) |
| **Respiratory** | | | Urinary tract infection | 29 (4.0) | 71 (2.0) |
| Lung edema | 3 (0.4) | 5 (0.1) | Infectious colitis | 4 (0.6) | 6 (0.2) |
| Atelectasis | 27 (3.8) | 46 (1.3) | Wound infection | 370 (51.5) | 41 (1.1) |
| Pneumonia | 341 (47.4) | 105 (2.9) | Wound disruption | 6 (0.8) | 9 (0.3) |
| Pyothorax | 0 (0) | 2 (0.1) | Decubitus | 32 (4.5) | 14 (0.4) |
| ARDS/respiratory failure | 18 (2.5) | 18 (0.5) | Hypothermia (<35°C) | 2 (0.3) | 4 (0.1) |
| Others | 5 (0.7) | 23 (0.6) | Drug allergy | 5 (0.7) | 2 (0.1) |
| **Coagulation** | | | Others | 20 (2.8) | 40 (1.1) |
| DIC/Coagulation disorder | 32 (4.5) | 31 (0.9) |  |  |  |
| Thrombopenia ^a^ | 12 (1.7) | 13 (0.4) |  |  |  |
| Others | 18 (2.5) | 9 (0.3) |  |  |  |

^a^ Thrombopenia is defined as platelets <50,000 mm^3^.

Definition of abbreviations: PE = pulmonary embolism; CSF = cerebrospinal fluid; CPA = cardiopulmonary arrest; ARDS = acute respiratory distress syndrome; DIC = disseminated intravascular coagulation; GI = gastrointestinal; MOF = multiple organ failure.

**Supplementary Table S3:** **Time to bone fixation of patients with and without pulmonary embolism**

|  | **n (%)** | |
| --- | --- | --- |
| **Time to bone fixation** | **PE group (n = 521)** | **Control group (n = 1,476)** |
| ≤24 h | 110 (22.4) | 359 (27.5) |
| >24 h, ≤48 h | 106 (21.6) | 161 (12.3) |
| >48 h, ≤72 h | 88 (17.9) | 172 (13.2) |
| >72 h, ≤96 h | 56 (11.4) | 110 (8.4) |
| >96 h, ≤120 h | 40 (8.1) | 94 (7.2) |
| >120 h, ≤144 h | 23 (4.7) | 90 (6.9) |
| >144 h | 68 (13.8) | 321 (24.6) |

Definition of abbreviations: PE = pulmonary embolism.
Missing data: 30 patients in the PE group and 169 patients in the control group.

**Supplementary Table S4:** **Association between pulmonary embolism and various characteristics among patients admitted to intensive care unit (sensitivity analysis)**

| **Variable** | **OR (95% CI)**  **(n = 1,910)** | **P value** |
| --- | --- | --- |
| Head AIS ≥ 3 | 0.76 (0.57–1.00) | 0.05 |
| Thorax AIS ≥ 3 | 1.18 (0.90–1.55) | 0.23 |
| Abdomen AIS ≥ 3 | 0.93 (0.62–1.38) | 0.70 |
| Spine AIS ≥ 3 | 1.62 (1.15–2.27) | 0.006 |
| Upper extremity AIS ≥ 3 |  |  |
| Long bone fracture |  |  |
| Open | 1.30 (0.80–2.10) | 0.29 |
| Closed | ^a^ |  |
| Lower extremity AIS ≥ 3 |  |  |
| Long bone fracture |  |  |
| Open | 2.11 (1.50–2.96) | <0.001 |
| Closed | 1.08 (0.74–1.58) | 0.69 |
| Blood transfusion | 0.84 (0.62–1.13) | 0.25 |
| Central vein catheter | 1.91 (1.27–2.86) | 0.002 |
| Any surgery | 3.53 (2.57–4.85) | <0.001 |

All models were adjusted for comorbidities that were present in ≥11 patients in both the pulmonary embolism and control groups.
The 3^rd^ model and the final model were adjusted for unclassifiable fracture in the lower extremity and pelvis.
^a^ Covariate that was not used for the model because it was present in <10 patients in the PE group.
Definition of abbreviations: OR = odds ratio; IQR = interquartile range; AIS = Abbreviated Injury Scale score.
